# Fast and accurate approximate inference of transcript expression from RNA-seq data

**DOI:** 10.1093/bioinformatics/btv483

**Published:** 2015-08-26

**Authors:** James Hensman, Panagiotis Papastamoulis, Peter Glaus, Antti Honkela, Magnus Rattray

**Affiliations:** ^1^Sheffield Institute for Translational Neuroscience (SITraN), Sheffield, UK,; ^2^Faculty of Life Sciences,; ^3^School of Computer Science, The University of Manchester, Manchester, UK and; ^4^Helsinki Institute for Information Technology (HIIT), Department of Computer Science, University of Helsinki, Helsinki, Finland

## Abstract

**Motivation:** Assigning RNA-seq reads to their transcript of origin is a fundamental task in transcript expression estimation. Where ambiguities in assignments exist due to transcripts sharing sequence, e.g. alternative isoforms or alleles, the problem can be solved through probabilistic inference. Bayesian methods have been shown to provide accurate transcript abundance estimates compared with competing methods. However, exact Bayesian inference is intractable and approximate methods such as Markov chain Monte Carlo and Variational Bayes (VB) are typically used. While providing a high degree of accuracy and modelling flexibility, standard implementations can be prohibitively slow for large datasets and complex transcriptome annotations.

**Results:** We propose a novel approximate inference scheme based on VB and apply it to an existing model of transcript expression inference from RNA-seq data. Recent advances in VB algorithmics are used to improve the convergence of the algorithm beyond the standard Variational Bayes Expectation Maximization algorithm. We apply our algorithm to simulated and biological datasets, demonstrating a significant increase in speed with only very small loss in accuracy of expression level estimation. We carry out a comparative study against seven popular alternative methods and demonstrate that our new algorithm provides excellent accuracy and inter-replicate consistency while remaining competitive in computation time.

**Availability and implementation:** The methods were implemented in R and C++, and are available as part of the BitSeq project at github.com/BitSeq. The method is also available through the BitSeq Bioconductor package. The source code to reproduce all simulation results can be accessed via github.com/BitSeq/BitSeqVB_benchmarking.

**Contact:** james.hensman@sheffield.ac.uk or panagiotis.papastamoulis@manchester.ac.uk or Magnus.Rattray@manchester.ac.uk

**Supplementary information:** Supplementary data are available at *Bioinformatics* online.

## 1 Introduction

RNA-seq is a technology with the potential to identify and quantify all mRNA transcripts in a biological sample (Mortazavi *et* *al.*, 2008). Some of these transcripts come from different isoforms or alleles of the same genes or from closely related homologous genes, and consequently they may share much of their primary sequence. Currently, popular RNA-seq technologies generate short reads that must be aligned to the genome or transcriptome to quantify expression levels. In some cases the observed reads could originate from several different transcripts and there may be few reads that are useful to distinguish these transcripts. It is therefore a challenging statistical problem to uncover the expression levels of closely related transcripts. A recent assessment confirms this by showing significant variability between results obtained using different computational pipelines (SEQC/MAQC-III Consortium, 2014).

Probabilistic latent variable models, in particular mixture models (Jiang and Wong, 2009; Glaus *et* *al.*, 2012; Katz *et* *al.*, 2010; Li and Dewey, 2011; Li *et* *al.*, 2010; Nariai *et* *al.*, 2013; Trapnell *et* *al.*, 2013; Turro *et* *al.*, 2011) provide a popular and effective approach for inferring transcript expression levels from RNA-seq data. Such models can be used to deconvolve the signal in the read data, assigning reads to alternative, pre-defined transcripts according to their probability of originating from each. The term mixture model derives from the interpretation of the data as being derived from a mixture of different transcripts, the mixture components, with each read originating from one component. Although reads originate from only one component they may map to multiple related components, resulting in some ambiguity in their assignment. Transcript expression levels are model parameters (mixture component proportions) that have to be inferred from the mapped read data. Due to their probabilistic nature these models can fully account for multiple mapping reads, complex biases in the sequence data, sequencing errors, alignment quality scores and prior information on the insert length in paired-end reads. Mixture models have been successfully applied to infer the proportion of different gene isoforms or allelic variants in a particular sample (Jiang and Wong, 2009; Katz *et* *al.*, 2010; Turro *et* *al.*, 2011), for inferring gene and isoform expression levels (Li *et* *al.*, 2010; Li and Dewey, 2011; Mortazavi *et al.*, 2008; Roberts and Pachter, 2013; Trapnell *et* *al.*, 2013) and for transcript-level differential expression calling (Glaus *et* *al.*, 2012; Trapnell *et* *al.*, 2013).

Inference in latent variable models such as these can be carried out by maximum likelihood (ML) or Bayesian parameter estimation. In ML the choice of parameters that maximizes the data likelihood is obtained through a numerical optimization procedure. In the case of mixture models a popular choice of algorithm is the Expectation Maximization (EM) algorithm, as first applied to this model and expressed sequence tag data by Xing *et* *al.* (2006) and later to RNA-seq data by Li *et* *al.* (2010). For Bayesian inference the most popular approach is Markov chain Monte Carlo (MCMC) and for the case of mixture models a Gibbs sampler is most often used (Glaus *et al.*, 2012; Katz *et* *al.*, 2010; Li and Dewey, 2011). An advantage of Bayesian inference is that one obtains a posterior probability over the model parameters rather than just a point estimate. This provides a level of uncertainty in the inferred transcript expression levels as well as information about the covariation between estimates for closely related transcripts. The uncertainty information can be usefully propagated into downstream analysis of the data, e.g. calling differentially expressed transcripts from replicated experiments (Glaus *et* *al.*, 2012).

A Bayesian method, BitSeq, was proposed in which inference was carried out using a collapsed Gibbs sampler (Glaus *et* *al.*, 2012). The method was shown to perform well, especially for the task of inferring the relative expression of different gene isoforms and for ranking transcripts according to their probability of being differentially expressed between conditions. However, for typical modern RNA-seq datasets with hundreds of millions of read-pairs the Gibbs sampler can be inconveniently slow, creating a computational bottleneck in applying a Bayesian approach. As the volume of data continues to grow and gene models are becoming more complex as more alternative transcripts are discovered, more efficient inference algorithms are required so that Bayesian methods can be used to provide practical computational tools.

An alternative approach to Bayesian inference is to use deterministic approximate inference algorithms such as Variational Bayes (VB) (reviewed in Bishop, 2006). While MCMC algorithms are attractive due to their asymptotic approximation guarantees, VB often provides a much faster method to obtain a good approximation to the posterior distribution. For models where Gibbs sampling can be applied there is typically a closely related VB Expectation Maximization (VBEM) algorithm. In this contribution, we show how VB can be used to massively speed up inference in the BitSeq model for transcript expression-level inference. We show that the mean transcript expression level estimates are very close to those obtained with MCMC. We use a recent formulation of VB (Hensman *et* *al.*, 2012) which is shown to provide a greater speed up when compared with a more standard VBEM algorithm. Our new algorithm is implemented in the most recent version of the BitSeq, allowing the method to be applied to much larger RNA-seq datasets in equal computing time.

An alternative VB method, TIGAR, was recently proposed for the same problem using a standard VBEM algorithm (Nariai *et* *al.*, 2013). The assumptions made in our approximation are similar to those used in TIGAR, but the empirical comparisons herein show that our proposed method performs better in terms of computation time and required memory, while also providing improved accuracy on real and simulated data. The improvement in terms of reduced computational cost is due to our adoption of a novel VB method. Furthermore, we investigate the effects of the variational assumption in this problem, and compare empirically to results using the gold standard, MCMC.

The article is organized as follows. In Section 2, we review the original BitSeq probabilistic model and describe our new inference algorithm, BitSeqVB, explaining the principles underlying our improved optimization scheme. In Section 3, we benchmark our new method against the original BitSeq algorithm and six popular alternative methods using realistic simulated data and real human RNA-Seq data. We consider accuracy in terms of expression estimation, relative with-gene transcript proportions and between-replicate consistency. We also compare the computation time required for all methods and compare the new VB algorithm to more standard MCMC and VBEM inference algorithms.

### 2 Methods

Our probabilistic model of RNA-seq follows Stage 1 of Glaus *et* *al.* (2012), and is similar to that used by RSEM. We summarize our notation in Table 1

**Table 1.** Summary of notations

*N*	Number of reads in the dataset
*M*	Number of transcripts in the transcriptome
$r_{n}$	The *n*th read
R	The collection of reads
T	The transcriptome
*T_m_*	The *m*th transcript
θ*_m_*	Proportion of transcript *T_m_* in the sample
*z_nm_*	Binary: *z_nm_* = 1 if read *n* comes from transcript *m*
$z_{n}$	Allocation vector of the *n*th read
Z	Collection of all allocation vectors
$\phi_{nm}$	Approximate posterior probability of *z_nm_* = 1
γ*_nm_*	Re-parameterization of $\phi_{nm}$

**Fig. 1.**
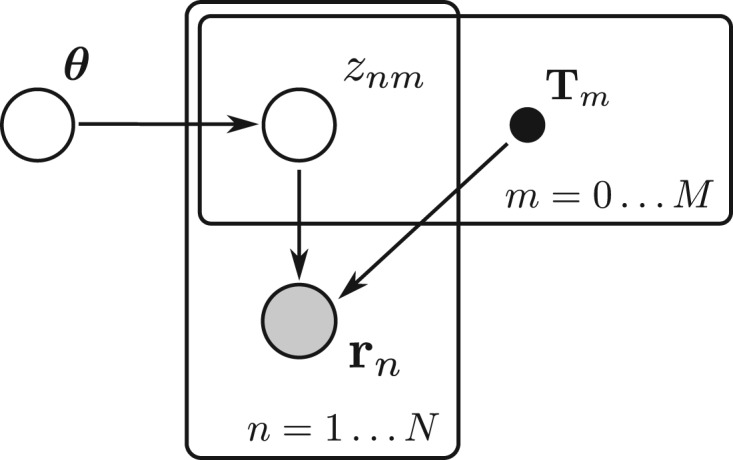
Graphical model of the RNA-seq mixture problem. Given a known Transcriptome T and some observed reads R, the inference problem is for θ through the latent variables Z

. The probabilistic model is shown using standard directed graphical notation in Figure 1. Here we have focused on the mixture part of the analysis, assuming that the model which associates reads to transcripts [i.e. $p(r_{n}\;|\;T_{m})$] is known. Following BitSeq (Glaus *et* *al.*, 2012), we compute this part of the model a priori, with parameters estimated from uniquely aligned reads. We consider RNA-seq assays independently, computing an approximate posterior for the transcript proportions θ in each assay. Subsequent analysis such as differential expression can be done using the estimated distributions of each assay.

### 2.1 The generative model

#### Transcript fragment proportions

The generative model for an RNA-seq assay is as follows. We assume that the experiment produces of collection of RNA fragments, where the abundance of fragments derived from transcript *T_m_* in the assay is θ*_m_.* Fragments are then sequenced in these proportions, so that the prior probability of any fragment corresponding to transcript *T_m_* is θ*_m_.* Introducing a convenient allocation vector $z_{n}$ for each read, we can write

(1) $$p(Z|\theta )=\prod_{n=1}^{N}\prod_{m=1}^{M}\theta_{m}^{z_{nm}},$$

where $z_{nm}\in \{0,1\}$ is a binary variable which indicates whether the *n*th fragment came from the *m*th transcript ($z_{nm}=1$) and is subject to $\sum_{m=0}^{M}z_{nm}=1$. We use Z to represent the collection of all allocation vectors. We note that both θ and Z are variables to be inferred, with θ the main object of interest. θ can be transformed later into some more convenient measure, for instance reads per kilobase of length per million sequenced reads (RPKM) (Mortazavi *et* *al.*, 2008), though it is more convenient from a probabilistic point of view to work with θ directly. The variables Z are sometimes known in the machine learning literature as latent variables. Although not of interest directly, inference of these variables is essential to infer θ.

#### Read model

An important part of the model is the likelihood term $p(r_{n}|T_{m})$ which is the probability of generating the *n*th read from the *m*th transcript. Writing the collection of all reads as $R={\left\{r_{n}\right\}}_{n=1}^{N}$, the likelihood given a set of alignments Z is

(2) $$p(R|T,Z)=\prod_{n=1}^{N}\prod_{m=1}^{M}p{\left(r_{n}|T_{m}\right)}^{z_{nm}},$$

where *T_m_* represents the *m*th transcript and T represents the transcriptome. The values of $p(r_{n}|T_{m})$ for all alignments can be computed before performing inference in θ since we are assuming a known transcriptome. For paired-end reads, the mates originate from a single fragment and their likelihood is inferred jointly. Denoting $r_{n}=(r_{n}^{\left(1\right)},r_{n}^{\left(2\right)})$, the likelihood of alignment is computed as

(3) $$P(r_{n}|T_{m})=P(l|T_{m})P(p|l,T_{m})\prod_{i=1,2}P(r_{n}^{\left(i\right)}|{\text{seq}}_{mlp})\;,$$

where *l* is the length of a fragment, *p* is its position and seq*_mlp_* denotes the underlying reference sequence. The fragment length distribution can be pre-defined or inferred empirically. The position likelihood, $P(p|l,T_{m})$, can be either uniform or account for different biases using an empirical model as in Glaus *et* *al.* (2012). The last term, $\prod_{i=1,2}P(r_{n}^{\left(i\right)}|{\text{seq}}_{mlp})$ describes the probability of observed read sequences based on quality scores and base discrepancy between read and reference. For detailed description of the alignment likelihood estimation please refer to Glaus *et* *al.* (2012).

#### Identifying noisy reads

Our model is similar to previous work (Glaus *et* *al.*, 2012), but does not contain a variable identifying reads as belonging to a ‘noise’ class. To circumvent the explicit formulation of a model with this variable, we introduce a ‘noise transcript’ which we append to the list of known transcripts. The generative probability of any read from this transcript, $p(r_{n}|T_{0})$, is again calculated according to the model described in Glaus *et* *al.* (2012). Due to the conjugate relationships between the variables in our model and those of Glaus *et* *al.* (2012), the models are the same, subject to a slight reformulation of the prior parameters.

#### Prior over θ

The final part of our model is to specify some prior belief in the vector θ. To make our approximations tractable, it is necessary to use a conjugate prior, which in this case is a Dirichlet distribution

(4) $$p(\theta )=\frac{\Gamma ({\hat{\alpha }}^{o})}{\prod_{m=1}^{M}\Gamma (\alpha_{m}^{o})}\prod_{m=1}^{M}\theta_{m}^{\alpha_{m}^{o}-1}$$

where $\alpha_{m}^{o}$ represents our prior belief in the values of θ*_m_* and ${\hat{\alpha }}^{o}=\sum_{m=1}^{M}\alpha_{m}^{o}$. We use a weak but proper prior $\alpha_{m}^{o}=1;\;m=0\ldots M$ which corresponds to a single ‘pseudo-count’ read (or read-pair) for each transcript.

### 2.2 Approximate inference

We are interested in computing the posterior distribution for the mixing proportions, $p(\theta \;|\;R,T)\propto \sum_{Z}p(R\;|\;T,Z)p(Z\;|\;\theta )p(\theta )$. For very small datasets, it is possible to perform exact Bayesian inference in this model, however for any realistically sized problem, exact inference is impossible due to the combinatorial explosion of the number of possible solutions. Our proposed solution is to use a collapsed version of Variational Bayes (VB). VB involves approximating the posterior probability density of all the model parameters with another distribution *q*,

(5) q(θ,Z)≈p(θ,Z|R,T).

The approximation is optimized by minimising the Kullback-Leibler (KL) divergence between q(θ,Z) and p(θ,Z|R,T) (Bishop, 2006). To make the VB approach tractable, some factorizations need to be assumed in the approximate posterior. In the case of the current model, we assume that the posterior probability of the transcript proportions factorizes from the alignments:

(6) q(θ,Z)=q(θ)q(Z).

Further factorizations in q(Z) occur due to the simplicity of the model, revealing $q(Z)=\prod_{n=1}^{N}q(z_{n})$. We write the approximate distribution for q(Z) using the parameters $\phi_{nm}$:

(7) $$q(Z)=\prod_{n=1}^{N}\prod_{m=1}^{M}\phi_{nm}^{z_{nm}}.$$

We need not introduce parameters for q(θ) since it will arise implicitly in our derivation in terms of ϕ.

#### The objective function

Approximate inference is performed by optimization: the parameters of the approximating distribution are changed so as to minimize the KL divergence. Whilst the KL divergence is not computable, it is possible to derive a lower bound on the marginal likelihood, maximization of which minimizes the KL divergence (see e.g. Bishop, 2006). Here we derive a lower bound which is dependent only on the parameters of q(Z), with the optimal distribution for q(θ) arising implicitly for any given q(Z). First we construct a lower bound on the conditional log probability of the reads R given the transcript proportions θ and the known transcriptome T:
(8)
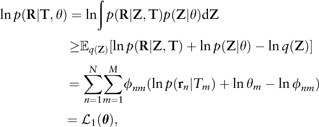

where the first line follows from Jensen’s inequality in a similar fashion to standard VB methods. We have denoted this conditional bound $L_{1}(\theta )$, which is still a function of θ. To generate a bound on the marginal likelihood, p(R | T), we need to remove this dependence on θ which we do in a Bayesian fashion, by substituting $L_{1}(\theta )$ into the following Bayesian marginalization:

(9) p(R|T)=∫p(R|T,θ)p(θ)dθ ≥∫exp{L1(θ)}p(θ)dθ.

Solving this integral and taking the logarithm gives us our final bound which equates to

(10) lnp(R|T)≥L=∑n=1N∑m=1Mϕnm(lnp(rn|Tm)−lnϕnm) +lnΓ(α^o)−lnΓ(α^o+N)−∑m=1M(lnΓ(αmo)−lnΓ(αmo+ϕ^m)),

where ${\hat{\phi }}_{m}=\sum_{n=1}^{N}\phi_{nm}$ and we also have that the approximate posterior distribution for θ is a Dirichlet distribution with parameters $\alpha_{m}^{o}+{\hat{\phi }}_{m}$.

### 2.3 Optimization

Having established the objective function as a lower bound on the marginal likelihood, all that remains is to optimize the variables of the approximating distribution q(Z,θ). The dimensionality of this optimization is rather high and potentially rather difficult. Optimization in standard VB is usually performed by an EM like algorithm, which performs a series of convex optimizations in each of the factorized variables alternately. In our formulation of the problem, we only need to optimize the parameters of the distribution q(Z), which we do by a gradient-based method. Taking a derivative of (10) with respect to the parameters ϕ gives

(11) $$\frac{\partial L}{\partial \phi_{nm}}=\text{ln}p(r_{n}\;|\;T_{m})-\text{ln}\phi_{nm}-1+\psi (\alpha_{m}^{o}+{\hat{\phi }}_{m}),$$

where *ψ* is the digamma function. To avoid constrained optimization we re-parameterize ϕ as *γ*:

(12) $$\phi_{nm}=\frac{{\text{e}}^{\gamma_{nm}}}{\sum_{m\prime =1}^{M}{\text{e}}^{\gamma_{nm\prime }}}$$

and it is then possible to optimize the variables γ using a standard gradient-based optimizer.

### 2.4 Geometry

Information geometry concerns the interpretation of statistical objects in a geometric fashion. Specifically, a class of probability distributions behaves as a Riemannian manifold with curvature given by the Fisher information. Amari (1998) showed that the direction of the steepest descent on such a manifold is given by the natural gradient:

(13) $$\tilde{\nabla }L=G^{-1}\nabla L\;,$$

where *G* is the Fisher information matrix. Since we are performing optimization of the distribution q(Z), we can make use of the natural gradient in computing a search direction (Honkela *et* *al.*, 2010). For our problem, we assume that the *N* × *M* matrix Z has been transformed into a *NM* vector, and the Fisher information corresponding to γ*_nm_*, $\gamma_{n\prime m\prime }$ is given by

(14) $$G[m,n,m\prime ,n\prime ]=\{\begin{array}{ll} \phi_{nm}-\phi_{nm}^{2}, & \text{if}\;n=n\prime \;\text{and}\;m=m\prime \\ -\phi_{nm}\phi_{nm\prime }, & \text{if}\;n=n\prime \;\text{but}\;m\neq m\prime \\ 0, & \text{otherwise}. \end{array}$$

We note that this structure is block-diagonal, and that each block can be easily inverted using the Sherman–Morrison identity, giving an analytical expression for $G^{-1}\nabla L$, and thus making the natural gradient very fast to compute (see Hensman *et* *al.* (2015) for more details). One can draw comparisons with a Newton method, where *G* would be replaced with a Hessian, though in the proposed case the system is much cheaper to compute.

The optimization of the variational parameters then proceeds as follows. Following random initialization, a unit step is taken in the natural gradient direction. Subsequent steps are subject to conjugate gradients (Honkela *et* *al.*, 2010). If the conjugate gradient step should fail to improve the objective we revert to a VBEM update, which is guaranteed to improve the bound. For more details, see Hensman *et* *al.* (2012).

### 2.5 Truncation

The optimization described above has *N* × *M* free parameters for optimization, one to align each read to each transcript. However, for most read-transcript pairs, $p(r_{n}\;|\;T_{m})$ will be negligibly small. We follow Glaus *et* *al.* (2012) in truncating the values of $p(r_{n}\;|\;T_{m})$ to zero for reads which do not suitably align. Examining the objective function (10) we see that we can also set $\phi_{nm}$ to zero for these truncated alignments (using the convention that 0ln(0)=0) and thus also $\gamma_{nm}=-\infty$ for the same. This truncation dramatically reduces the computational load of our algorithm, reducing the dimensionality of the optimization space as well as reducing the number of operations needed to compute the objective.

### 2.6 The approximate posterior

Having fitted our model, we may wish to propagate the posterior distribution through a second set of processing, for example to identify differentially expressed transcripts as in BitSeq stage 2 (Glaus *et* *al.*, 2012). Whilst it may be desirable to solve both stages together in a Bayesian framework, the size of the problem generally forbids this, therefore we propose the use of either a moment-matching or sampling procedure to propagate q(θ) through further analysis. The approximate posterior q(θ) is a Dirichlet distribution, whose marginals have the following useful properties:

(15) $$E\left[\theta_{m}\right]=\frac{\alpha_{m}^{o}+{\hat{\phi }}_{m}}{{\hat{\alpha }}^{o}+N},$$

(16) $$\text{var}[\theta_{m}]=(\alpha_{m}^{o}+{\hat{\phi }}_{m})({\hat{\alpha }}^{o}+N-\alpha_{m}^{o}-{\hat{\phi }}_{m})C,$$

(17) $$\text{cov}[\theta_{m},\theta_{m}\prime ]=-(\alpha_{m}^{o}+{\hat{\phi }}_{m})(\alpha_{m\prime }^{o}+{\hat{\phi }}_{m\prime })C,$$

with $C={\left({\hat{\alpha }}^{o}+N\right)}^{-2}{\left({\hat{\alpha }}^{o}+N+1\right)}^{-1}$. This approximate posterior is somewhat inflexible, in that it cannot express arbitrary covariances between the transcripts. This arises from the factorizing assumption amongst the assignment of reads to transcripts: reads are assigned independently in the variational method and their dependence cannot be modelled. This is reflected in the results section where we show empirically that the VB approximation leads to an underestimation of the variance. Nonetheless, this simplifying assumption leads to very accurate expression estimates much faster than MCMC.

## 3 Results and discussion

The proposed BitSeqVB algorithm was compared with Cufflinks (Trapnell *et* *al.*, 2010), RSEM as well as the corresponding MCMC sampler RSEM-PME (Li and Dewey, 2011), BitSeqMCMC (Glaus *et* *al.*, 2012), eXpress (Roberts and Pachter, 2013), Casper (Rossell *et* *al.*, 2014), Sailfish (Patro *et* *al.*, 2014), Tigar2 (Nariai *et* *al.*, 2014) and Kallisto (Bray *et* *al.*, 2015). We note that both MCMC samplers (RSEM-PME and BitSeqMCMC) use similar collapsed Gibbs algorithms but are initialized differently: RSEM-PME starts from the ML solution found by RSEM while BitSeqMCMC starts from a random initialization and therefore requires more iterations to find a good solution.

We used two main ways for benchmarking: analysis on synthetic data allowed comparison with a known ground truth under a variety of generative scenarios; analysis on high-quality replicated human data focused on inter-replicate consistency following the evaluation of Rossell *et* *al.* (2014). We find BitSeqVB to have excellent inter-replicate consistency and accuracy, closely approximating the original MCMC algorithm, while also being competitive with other methods in terms of run-time. We subsequently analyze in more detail the approximation to the posterior used in the BitSeqVB method. For comparison with other methods, we used default settings where appropriate: both MCMC sampling methods use 1000 posterior samples as default. However, this number refers to effective samples (Gelman *et* *al.*, 2003) in BitSeqMCMC and not to single iterations as in RSEM-PME. We turned off creating of unnecessary output files in RSEM. The experiments were conducted on a four core workstation. All the details of the experiments can be found at the aforementioned URL.

### 3.1 Inference accuracy on synthetic data

RNA-seq reads from *M* = 48 009 transcripts of the UCSC/hg19 transcriptome annotation (Kent *et* *al.*, 2002) were simulated using the Spanki software (Sturgill *et* *al.*, 2013). The expression is evaluated in three different measures: transcript expression accuracy (Theta), transcript within-gene relative proportion accuracy (WGE-True) and inter-replicate consistency (WGE-Inter). The first two measures (Theta and WGE-TRUE) compare the resulting estimates against the ground-truth. On the other hand, WGE-Inter compares the consistency of within-gene estimates across independent repetitions of the same experiment. This implies that an algorithm yielding constant estimates independent of any data could achieve WGE-Inter=0, but it would obviously do very poorly on WGE-True. Thus, a good score on WGE-Inter is necessary but not sufficient for a method to perform well in practice. For further details of the evaluation measures see supplementary material (Section 5).

A ground truth was generated using four different models of transcript expression, according to the following scenarios:

1. estimated expression levels from real data using BitSeqMCMC (≈56 million reads per replicate)
2. randomly selected expression levels according to a uniform distribution defined on the set (10, 200) (≈7.8 million reads per replicate)
3. a high-dimensional mixture of Poisson Generalized Linear models, which was recently used to model the heterogeneity in RNA-seq datasets (Papastamoulis *et* *al.*, 2014b) (≈5.5 million reads per replicate)
4. estimated expression levels from real data using RSEM (≈18 million reads per replicate)

For each scenario five replicates are generated according to a Negative Binomial model. Full details of the four scenarios are described in the Supplementary Material. Finally, the resulting reads-per-kilobase (RPK) values were fed into Spanki. Next, the simulated reads were aligned to the reference annotation using Bowtie2 and/or Tophat2. In particular, BitSeq, RSEM, eXpress and Tigar require transcriptomic alignments so Bowtie2 (version 2.0.6) (Langmead and Salzberg, 2012) was used, while Cufflinks and Casper work with genomic alignments using Tophat2 and Bowtie2. On the other hand, Sailfish and Kallisto produce their own alignments using *k*-mers mapping and pseudo-alignments, respectively. The corresponding mapping rate for genomic or transcriptomic alignments was 96%. The same amount of reads pseudo-aligned when using Kallisto, whilst Sailfish mapped a smaller portion of *k*-mers (≈63%).

**Fig. 2.**
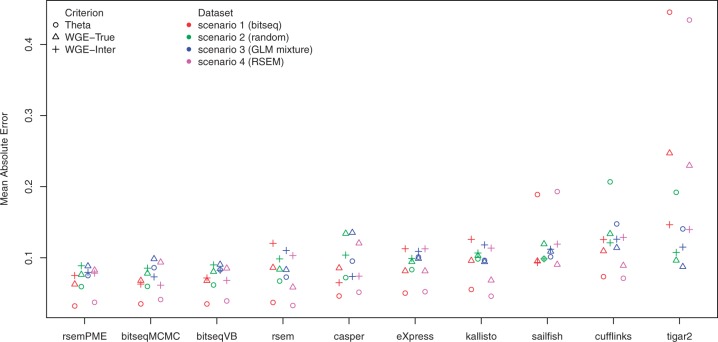
Ranking of methods for five replicates of simulated RNA-seq reads. WGE-Inter: inter-replicate consistency of within gene estimates, WGE-True: within gene estimates compared with the true values and Theta: estimated relative transcript expression compared with the true values. Scores have been normalized to unity per dataset. Alternative normalizations are available in supplementary material (Supplementary Fig. S9)

Figure 2 displays the mean absolute error (MAE) according to the three criteria, after performing the following normalization:

$$\sum_{m\in \text{methods}}{\text{MAE}}_{m}^{\left(c\right)}=1,$$

**Fig. 3.**
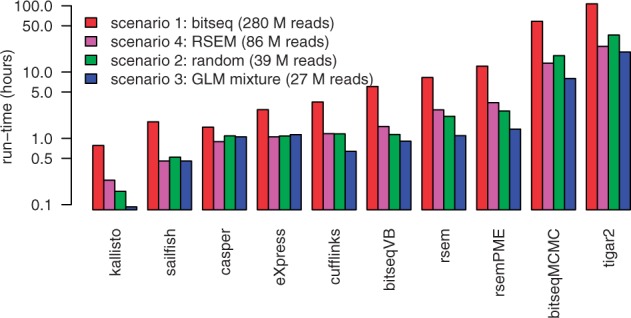
Run-time in hours (log-scale) for four synthetic data samples with five replicates per sample. The total number of simulated reads is shown in parenthesis

∀c∈{Theta,WGE-Inter,WGE-True}, to make all criteria equally weighted for each scenario. Moreover, the ‘Theta’ and ‘WGE-True’ metrics were averaged across the five replicates, while ‘WGE-Inter’ was averaged across all ten combinations of pairs of replicates. The methods were ranked with respect to their average across the three criteria. RSEM-PME, BitSeqMCMC and BitSeqVB are ranked as best when considering all three criteria. RSEM has similar accuracy in terms of the ground truth expression (Theta and WGE-True) but has lower inter-replicate consistency (WGE-Inter). Conversely, Casper achieves good performance with respect to inter-replicate consistency (WGE-Inter) but is less accurate in comparison to the ground truth values (WGE-True and Theta). The ranking of methods with respect to run-time is shown in Figure 3. Note that the run-time calculation excludes the alignment procedure, but includes all other computations (including computing alignment probabilities in BitSeq’s case). An exception is made for Sailfish and Kallisto, where alignment is not required, making these by far the fastest methods. Timings which include the time required for alignment are provided in Supplementary Figure S12.

**Fig. 4.**
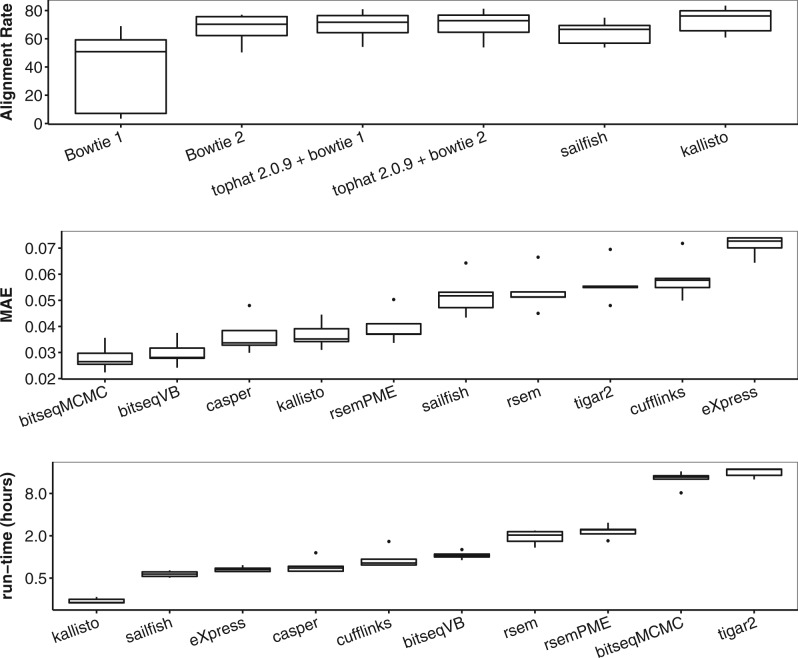
Five ENCODE pairs of replicates. (**a**) Alignment rates for transcriptome mapping (Bowtie1 and Bowtie2), genome mapping (Tophat 2.0.9 with Bowtie1 and Bowtie2), k-mers mapping (Sailfish) and pseudo-alignments (Kallisto). (**b**) Ranking of methods in terms of the Mean Absolute Error. (**c**) Run-time in hours (log-scale) with 24.6 M (mapped) reads per sample

The plots of inter-replicate consistency between pairs of replicates are shown in the supplementary material (Figs. 2, 4, 6 and 8). As seen there, Kallisto, RSEM, Sailfish, Tigar2, Cufflinks and eXpress, produce estimates close to the boundary of the parameter space. This is also obtained for RSEM-PME except for scenario 2. This behaviour is avoided when using BitSeqMCMC, BitSeqVB and Casper.

The accuracy of BitSeqVB is very close to the two sampling methods BitSeqMCMC and RSEM-PME, but it is consistently faster that these approaches, being about 10 times faster than BitSeqMCMC and 2 times faster than RSEM-PME on average (RSEM-PME is significantly faster than BitSeqMCMC because is uses many fewer iterations of MCMC). BitSeqVB has similar speed to the Cufflinks method in most cases whilst exhibiting much better accuracy.

We conclude that the proposed VB algorithm is competitive in speed while exhibiting both high accuracy and good inter-replicate consistency.

### 3.2 Replicate consistency in human data

A recent study (Rossell *et* *al.*, 2014) used the mean absolute error between pairs of replicates of the same ENCODE experiment to assess the accuracy of transcript expression estimation methods. For this purpose, the relative within gene expression estimates are used (WGE-Inter). Here, we provide an extended version of this analysis to benchmark against BitSeqMCMC and six other methods.

In total, five ENCODE datasets (Tilgner *et* *al.*, 2012) consisting of 2 × 76 bp reads were selected, corresponding to the following pairs of replicates: (SRR307897, SRR307898), (SRR307901, SRR307902), (SRR307907, SRR307908), (SRR307911, SRR307912), (SRR307915, SRR307916). All methods were applied assuming the same UCSC/hg19 transcriptome annotation as in the previous section. According to the alignment rates shown in Figure 4a, all methods work with almost the same number of mapped reads when Bowtie2 is used. This is not the case for Bowtie1 which for some reason fails on this dataset.

**Fig. 5.**
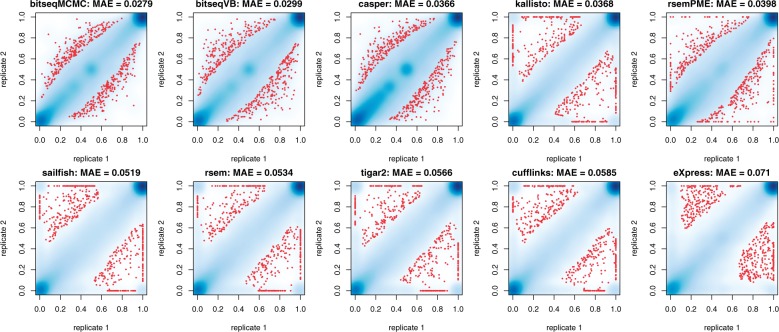
Scatterplots of within gene estimates for one pair of replicates (SRR307907 and SRR307908) from the ENCODE data. The blue color corresponds to a smoothed color density representation of the scatterplot

Figure 4b illustrates the ranking of methods in terms of the MAE criterion, averaged across the five datasets. We conclude that BitSeqMCMC has best inter-replicate consistency, closely followed by BitSeqVB, while Casper comes next. Sailfish, RSEM, Tigar2 and Cufflinks exhibit almost two times larger MAE, while eXpress is almost 2.5 times worse according to this measure. Based on these five samples there is a partial order: BitSeqMCMC≻BitSeqVB≻{Casper, Kallisto}≻RSEM-PME≻{RSEM, Sailfish}≻{Cufflinks, Tigar2}≻eXpress, where ≻ denotes ‘is better in every experiment’. Excluding BitSeq (MCMC and VB) and Casper, we see that many methods produced estimates close to the boundary of the parameter space, as seen in Figure 5. This means that many transcripts are estimated as weakly or non-expressed in one replicate while being highly expressed in the other. This problem appears to affect methods using ML estimation (RSEM, Sailfish, Cufflinks, eXpress) or Bayesian methods using a very weak prior (Tigar2). Casper ensures consistency with a strong prior, but this may degrade the accuracy of absolute estimates relative to BitSeq because of stronger regularization. We note that Casper uses MAP parameter estimation, finding the mode of the posterior distribution, while the BitSeq methods estimate the mean of the posterior distribution. Using the posterior mean may avoid spurious values where the mode is a long way from the mass of the posterior without the need for an overly strong prior. Finally, note that the coherency of inter-replicate consistency estimates in our simulation study (Supplementary Figs S2, S4, S6 and S8) with the one reported here.

The run-time for each method is displayed in Figure 4c. BitSeqVB is comparable to the fastest methods (except for Kallisto which is by far the fastest method) while being ranked as second in terms of the MAE criterion. We conclude that BitSeqVB offers perhaps the best trade-off in accuracy and runtime on these datasets.

Finally, we mention that the BitSeqMCMC performance here is in stark contrast with the performance reported in Rossell *et* *al.* (2014). The reason for this is that in Rossell *et* *al.* (2014) reads were aligned using Bowtie1 whereas we are using Bowtie2. As seen in Figure 4a, Bowtie1 can exhibit very low alignment rates for these samples. Interestingly, this behaviour is not present when Bowtie1 is combined with Tophat for genome mapping. The low alignment rates of Bowtie1 means that methods have available only a tiny fraction of the useful data, leading to less accurate results. This explains the weak agreement of same transcript estimates between pairs of replicates reported for BitSeq in Rossell *et* *al.* (2014) and is a reminder that it is very important to check the alignment rates.

### 3.3 Analysis of the variational Bayes approximation

To examine the properties of the variational approximation, we focused on ENCODE dataset SRR307907 (Tilgner *et* *al.*, 2012). This contained 30.8 million reads, each 76 bp. The reads were again mapped to the same UCSC/hg19 reference transcriptome resulting in 23.7 million mapped reads.

**Fig. 6.**
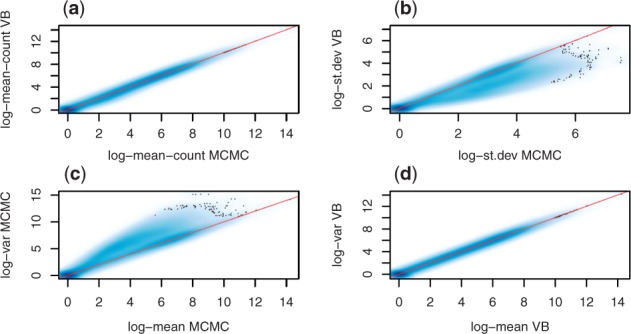
A comparison of the first two moments of the approximate posterior expression in counts per transcript: (**a**) posterior mean (*R*^2^ correlation is 0.999) (**b**) posterior standard deviation: the VB method significantly under-estimates the posterior variance ($\sigma^{2}$). (**c**), (**d**) posterior mean-variance relationship in MCMC and VB respectively. Shading represents the number of transcripts in each region

Our main potential concern in using the VB method is the quality of approximation to the posterior. Figure 6a shows a comparison of the variational posterior with a ground truth computed by MCMC with a very large sampling time. We conclude that the VB method consistently provides very accurate estimates of the posterior mean across the whole range of expression levels. The estimates of posterior variance are less consistent and for a fraction of transcripts the variances are underestimated (Fig. 6b). It appears that VB only estimates the Poisson variance associated with random sampling of reads (Fig. 6d), whereas the true posterior variance is larger for some transcripts due to the uncertainty in assigning multi-mapping reads (Fig. 6c). If estimation of the expression level is all that is required, then it would seem that the VB method suffices. However, downstream methods which make use of uncertainty in the transcript quantification [such as the differential expression analysis proposed in BitSeq stage 2 (Glaus *et* *al.*, 2012)] may suffer from the poor approximation in terms of posterior variance. This can potentially be addressed by augmenting the VB method with a more accurate approximation as done in a recent study that proposed a new VB algorithm with improved variance estimates and a tighter lower bound on the log-marginal likelihood (Papastamoulis *et* *al.*, 2014a).

### 3.4 Convergence comparison

We further investigate convergence properties of MCMC and VB in terms of mean expression. RNA-seq data was obtained from ENCODE experiment SRX110318, run SRR387661, generating 124.8 million 76 bp read-pairs. We mapped the reads using Bowtie 2 to a reference transcriptome using 8713 transcripts of chromosome 19 from Ensembl human cDNA, release 70 (Flicek *et* *al.*, 2013).

**Fig. 7.**
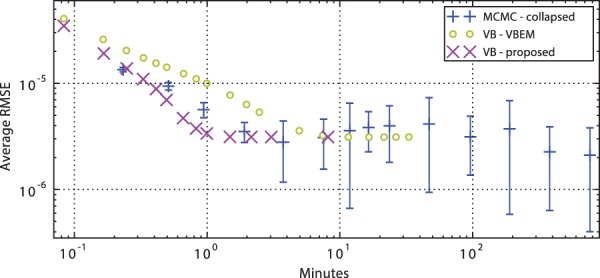
Convergence comparison of Collapsed MCMC with standard VB algorithm and VB with Fletcher-Reeves conjugate gradient optimization. Expression estimates obtained by very long run of MCMC are used as a ground truth and average root mean square error over 10 runs was calculated, two standard deviations are used as error bars. The VB methods with several randomized initial conditions showed negligible differences in convergence

As the true expression levels are unknown, we used a long run of MCMC as the ground truth for mean expression estimates. Running the inference methods for a certain number of iterations, we record the run time and calculate Root Mean Square Error (RMSE) of estimated expression. The convergence of our variational method (BitSeqVB) and the original Gibbs sampling procedure (BitSeqMCMC) is shown in Figure 7. We also include a standard implementation of VB (similar to Nariai *et* *al.* (2013)) but using the BitSeq model (denoted VBEM). It is straightforward to derive this algorithm from our VB algorithm derivation since standard VBEM is obtained as a special case of steepest descent VB learning (Hensman *et* *al.*, 2012). Our implementation of VB converges first in about 2 min. Surprisingly, some runs of collapsed MCMC converge to better estimates even faster than standard VB, which takes around 10 min. However, as MCMC is a stochastic method, an estimate that is consistently better than the results obtain by VB is only obtained after 900 min.

## 4 Conclusion

We have presented a new Variational Bayes method for inference of transcript expression from RNA-seq data. Building on previous work in BitSeq, we have presented a fast approximate inference method. The mean of the posterior distribution of expression levels was very well estimated in substantially less time than the original MCMC algorithm. The method is therefore suitable when point estimates of expression are sufficient, especially if time and computational resources are limited. We have compared both the original BitSeq algorithm and our new method with the majority of available methods for transcript expression estimation and conclude that BitSeqVB is highly competitive both in terms of expression estimation and run-time. We also note that an existing VBEM algorithm implementation, TIGAR, does not provide a significant improvement over Gibbs sampling in terms of computational time in our examples, as well as having a very high memory requirement.

The newest method considered here, Kallisto, is found to be extremely fast and perform with very good accuracy compared with other ML approaches. This speed-up is achieved through avoiding full alignment and simplifying the likelihood computation through using a pseudo-alignment approach. However, the method still produces estimates at the boundary in our between-replicate comparisons similar to all ML methods. It would therefore be very interesting to apply a Bayesian algorithm, such as the fast VB method proposed here, using the same likelihood model as Kallisto.

Finally, we suggest some areas for future development. The fast and consistent convergence of the VB method makes it useful for quick examination of the data before the Gibbs sampler is run. Further, since it provides an excellent approximation to the mean of the posterior, it could be used to e.g. reduce the burn-in time for the Gibbs sampler, or as the initial stage of a more sophisticated approximating technique, as in Papastamoulis *et* *al.* (2014a).

## Supplementary Material

Supplementary Data
