# Supplementary material for: Fast and accurate approximate inference of transcript expression from RNA-seq data
Source: Bioinformatics. 2015 Aug 26;31(24):3881–9. doi: 10.1093/bioinformatics/btv483 (PMC4673974; doi:10.1093/bioinformatics/btv483)
Supplement: Supplementary Data [file supp_31_24_3881__index.html]

Fast and accurate approximate inference of transcript expression from RNA-seq data — Fast and accurate approximate inference of transcript expression from RNA-seq data — Supplementary Data 

# Fast and accurate approximate inference of transcript expression from RNA-seq data

## Supplementary Data

files

- Supplementary Data - pdf file
