# Supplementary material for: Fast and accurate approximate inference of transcript expression from RNA-seq data
Source: Bioinformatics. 2015 Aug 26;31(24):3881–9. doi: 10.1093/bioinformatics/btv483 (PMC4673974; doi:10.1093/bioinformatics/btv483)
Supplement: Supplementary Data [file supp_btv483_supplemental-bioinformatics.pdf]

BIOINFORMATICS

Vol. 00 no. 00 2015
Pages 1–4

Supplementary material for the article: "Fast and accurate approximate inference of transcript expression from RNA-seq data"

James Hensman [1 †*], Panagiotis Papastamoulis [2 †*], Peter Glaus [3], Antti Honkela [4] and Magnus Rattray [2 †]

[1]Sheffield Institute for Translational Neuroscience (SITraN), Sheffield, UK
[2]Faculty of Life Sciences, The University of Manchester, Manchester, UK
[3]School of Computer Science, The University of Manchester, Manchester, UK
[4]Helsinki Institute for Information Technology HIIT, Department of Computer Science, University of Helsinki, Helsinki, Finland

We describe the generative process of the RPK values used in the simulation section of the manuscript. Let $\mathcal{NB}(\mu, \phi)$ denote the Negative Binomial distribution, with mean equal to μ and variance equal to $\mu + \mu^2/\phi$, $\mu \geqslant 0$, $\phi > 0$. Denote by RPK_{jm} the RPK value for transcript $m = 1, \ldots, M$ at replicate $j = 1, \ldots, J$, where J and M denote the number of replicates and the total number of transcripts, respectively.

1 SCENARIO 1: BITSEQ ESTIMATES FROM REAL DATA

We used human data (SRR307907) from the ENCODE project in order to capture the dynamics of realistic RNA-seq datasets. BitSeq (MCMC) was used to estimate the relative expression levels of $M = 48009$ transcripts. Next, the resulting estimates was used as input to generate the baseline mean. Reads were simulated according to the following generative process:

$$\mathrm{RPK}_{jm} \sim \mathcal{NB}(\widehat{\mu}_m, 50), \quad m = 1, \ldots, M, j = 1, \ldots, 5.$$

with $\widehat{\mu}_m$, $m = 1, \ldots, M$ denoting the corresponding estimates of RPK values according to BitSeq MCMC. This resulted in 56 million paired-end reads of 76 base-pairs per replicate, almost 280 million reads in total.

Figures 1 and 2 illustrate the scatterplots of within gene estimates for the first replicate versus the true values and the second replicate estimates, respectively. The plots are ordered according to the inter-replicate consistency Mean Absolute Error, as shown in Figure 2. Note that BitSeqMCMC, BitSeqVB and Casper are the only methods which avoid extreme outliers on the boundary of the inter-replicate consistency graphs.

2 SCENARIO 2: RANDOM SELECTION

A ground truth was generated using randomly selected levels of transcript expression, and the resulting reads-per-kilobase (RPK) values were fed into Spanki. Let $\mathcal{U}(\alpha, \beta)$ denotes the uniform distribution defined on the set (α, β), $\alpha < \beta$. Reads were simulated according to the following generative process:

$$\begin{aligned} \mu_m &\sim \mathcal{U}(10, 200), \quad m = 1, \ldots, M \\ \mathrm{RPK}_{jm} &\sim \mathcal{NB}(\mu_m, 20), \quad m = 1, \ldots, M, j = 1, \ldots, 5. \end{aligned}$$

This resulted in 15.8 million paired-end reads of 76 base-pairs per replicate, almost 80 million reads in total.

Figures 3 and 4 illustrate the scatterplots of within gene estimates for the first replicate versus the true values and the second replicate estimates, respectively. The plots are ordered according to the inter-replicate consistency Mean Absolute Error, as shown in Figure 4. Note that BitSeqMCMC and BitSeqVB are the only methods which avoid extreme outliers on the boundary of these graphs.

3 SCENARIO 3: MIXTURE OF POISSON GLMS

A ground truth was generated using a mixture of Poisson Generalized Linear models. It has been recently demostrated that this approach can be used in order to model the underlying heterogeneity in RNA-seq datasets (?). Let $\mathcal{P}(\lambda)$ denotes the Poisson distribution with mean $\lambda > 0$ and let also $\mathcal{D}(\alpha_1, \ldots, \alpha_K)$ denotes the Dirichlet distribution with $\alpha_k > 0$, $k = 1, \ldots, K$ for a given positive integer K. Reads were simulated according to the following generative process:

$$\begin{aligned} K &= 20 \\ (\pi_1, \ldots, \pi_K) &\sim \mathcal{D}(1, \ldots, 1) \\ x_m &\sim \mathcal{U}(0, 30) \\ \log \lambda_{jkm} &= \alpha_k + \beta_{jk} x_m \\ (\mu_{1m}, \ldots, \mu_{5m}) &\sim \sum_{k=1}^{K} \pi_k \prod_{j=1}^{5} \mathcal{P}(\lambda_{jkm}), \\ \mathrm{RPK}_{jm} &\sim \mathcal{NB}(\mu_{jm}, 50), \end{aligned}$$

*these authors contributed equally

†to whom correspondence should be addressed

© Oxford University Press 2015.

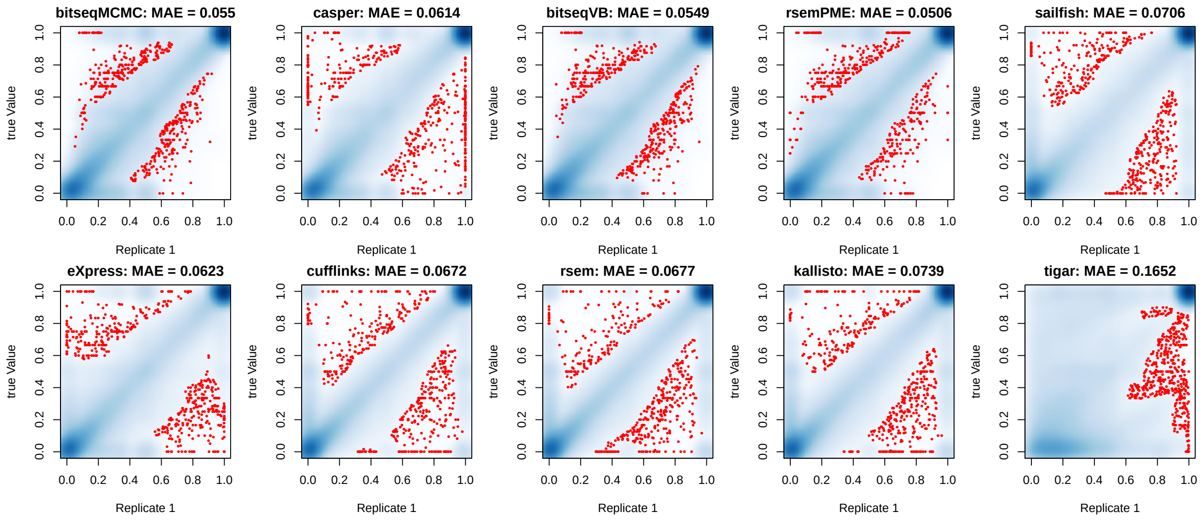

Fig. 1. Scenario 1: Scatterplots of within gene estimates for one replicate of the simulated data versus the true values. The blue color corresponds to a smoothed color density representation of the scatterplot and the red color emphasizes points from the lowest regional densities.

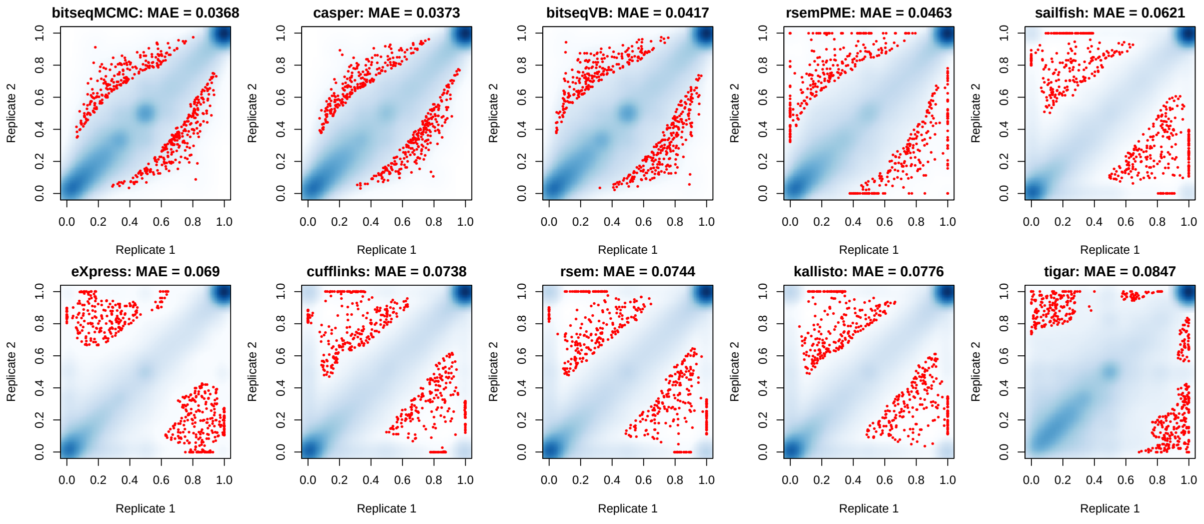

Fig. 2. Scenario 1: Scatterplots of within gene estimates for one pair of replicates from the simulated RNA-seq reads. The blue color corresponds to a smoothed color density representation of the scatterplot and the red color emphasizes points from the lowest regional densities.

where $m = 1, \ldots, M$, $j = 1, \ldots, 5$ and $k = 1, \ldots, K$. The regression coefficients (α_k, β_{jk}) are generated as follows.

$$\alpha_k = \frac{K^* - k}{2}, \quad k = 1, \ldots, K,$$

$$\beta_{jk} = \begin{cases} \frac{10-\alpha_k}{10}, & k = 1, \ldots, K,\ j = 1 \\ \beta_{1k} + \varepsilon_{jk}, & j \geqslant 2,\ \varepsilon_{jk} \sim \mathcal{N}(0, 0.001^2) \end{cases}$$

where $K^* := \lfloor 0.5 + (K+1)/2 \rfloor$ ($\lfloor \alpha \rfloor$ stands for the integer part of α).

This resulted in 5.5 million paired-end reads of 76 base-pairs per replicate, almost 27.5 million reads in total. Figures 5 and 6 illustrate the scatterplots of within gene estimates for the first replicate versus the true values and the second replicate estimates, respectively. The plots are ordered according to the inter-replicate consistency Mean Absolute Error, as shown in Figure 6. Note that BitSeqMCMC, BitSeqVB and Casper are the only methods which avoid extreme outliers on the boundary of the inter-replicate consistency graphs.

4 SCENARIO 4: RSEM ESTIMATES FROM REAL DATA

We used human data (replicates SRR307907 and SRR307908) from the ENCODE project in order to capture the dynamics of realistic RNA-seq datasets. RSEM was used to estimate the relative expression levels of $M = 48009$ transcripts for each dataset. Next,

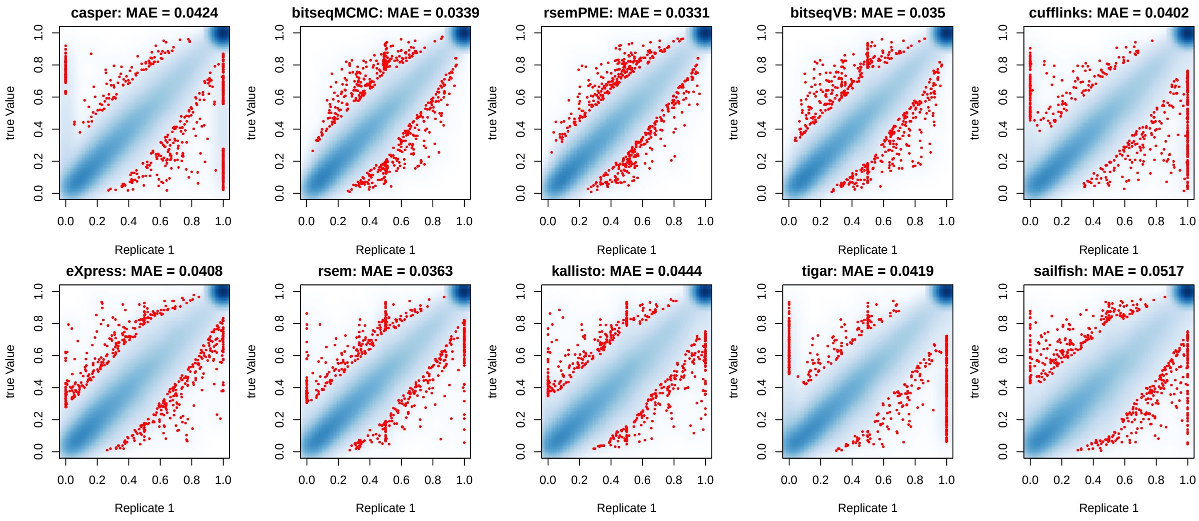

Fig. 3. Scenario 2: Scatterplots of within gene estimates for one replicate of the simulated data versus the true values. The blue color corresponds to a smoothed color density representation of the scatterplot and the red color emphasizes points from the lowest regional densities.

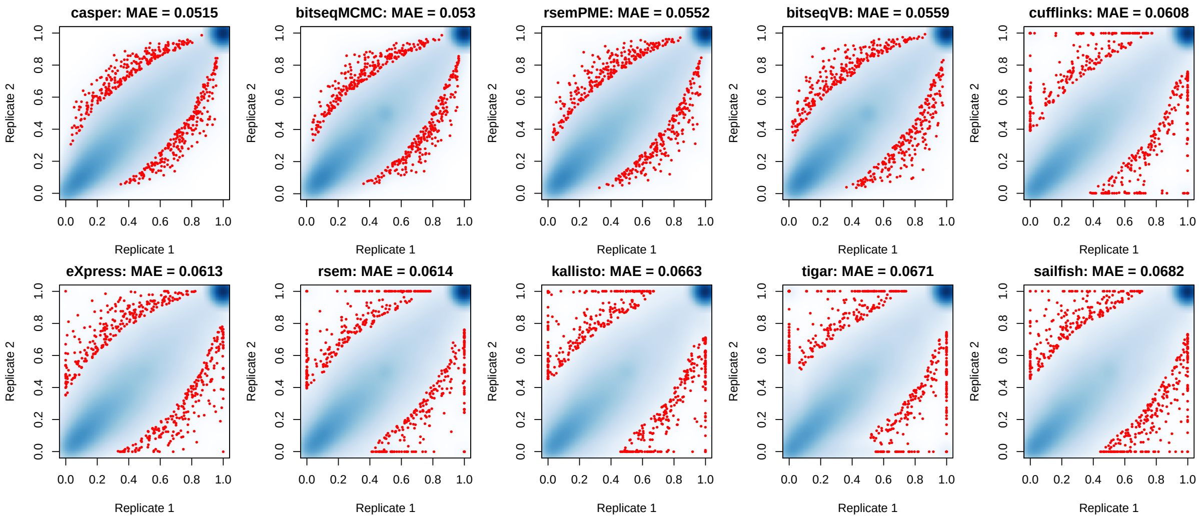

Fig. 4. Scenario 2: Scatterplots of within gene estimates for one pair of replicates from the simulated RNA-seq reads. The blue color corresponds to a smoothed color density representation of the scatterplot and the red color emphasizes points from the lowest regional densities.

the resulting estimates were averaged and used as input to generate the baseline mean. Reads were simulated according to the following generative process:

$$\text{RPK}_{jm} \sim \mathcal{NB}(\widehat{\mu}_m, 50), \quad m = 1, \ldots, M, j = 1, \ldots, 5.$$

with $\widehat{\mu}_m$, $m = 1, \ldots, M$ denoting the corresponding estimates of RPK values according to RSEM. This resulted in 18 million paired-end reads of 76 base-pairs per replicate, almost 86 million reads in total.

Figures 7 and 8 illustrate the scatterplots of within gene estimates for the first replicate versus the true values and the second replicate estimates, respectively. The plots are ordered according to the inter-replicate consistency Mean Absolute Error, as shown in Figure 8. Note that BitSeqMCMC, BitSeqVB and Casper are the only methods which avoid extreme outliers on the boundary of the inter-replicate consistency graphs.

The overall ranking of methods is shown in Figure 9. Now we used an alternative normalisation by dividing each score by the minimum per criterion, so that the normalised score of the best method is equal to 1.

5 EVALUATION MEASURES

The following measures are used.

Theta: $\frac{1}{J}\sum_{j=1}^{J}\frac{1}{M}\sum_{m=1}^{M}|\log\widehat{\theta}_m^{(j)} - \log\theta_m^{(j)}|$, where $\theta_m^{(j)}$ and $\widehat{\theta}_m^{(j)}$ denote true and estimated relative transcript expression for transcript m of replicate j, $m = 1, \ldots, M$, $j = 1, \ldots, J$.

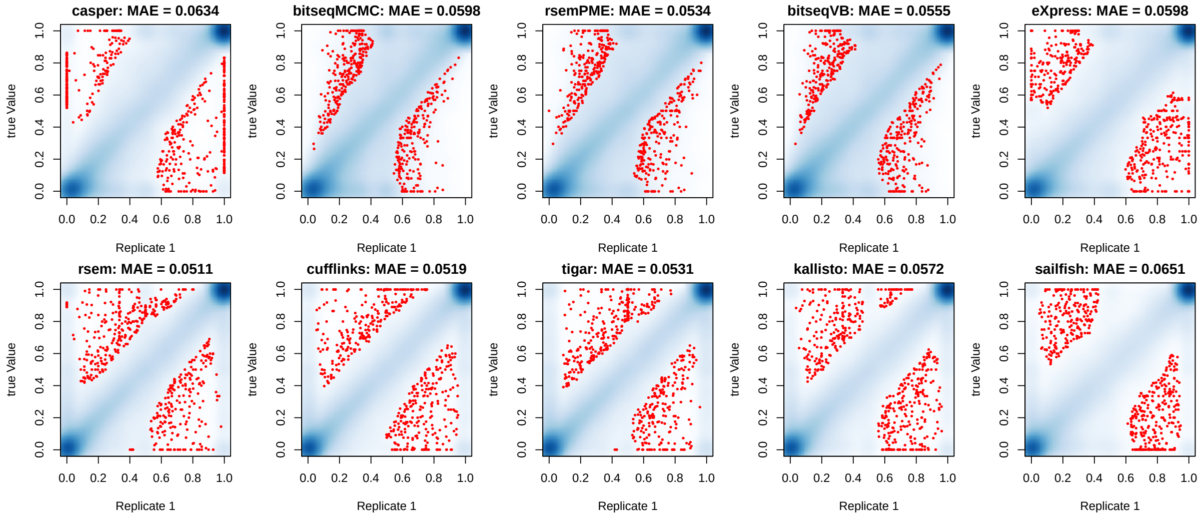

Fig. 5. Scenario 3: Scatterplots of within gene estimates for one replicate of the simulated data versus the true values. The blue color corresponds to a smoothed color density representation of the scatterplot and the red color emphasizes points from the lowest regional densities.

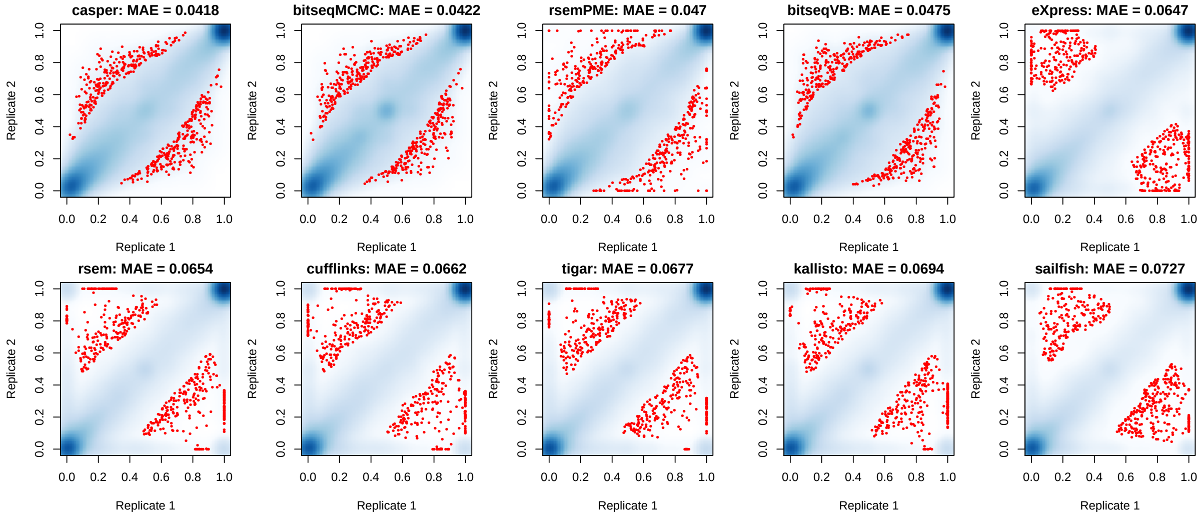

Fig. 6. Scenario 3: Scatterplots of within gene estimates for one pair of replicates from the simulated RNA-seq reads. The blue color corresponds to a smoothed color density representation of the scatterplot and the red color emphasizes points from the lowest regional densities.

WGE-True: $\frac{1}{J}\sum_{j=1}^{J}\frac{1}{M}\sum_{m=1}^{M}|\widehat{q}_m^{(j)} - q_m^{(j)}|$, where $q_m^{(j)} := \frac{\theta_m^{(j)}}{\sum_{k\in T_m}\theta_m^{(j)}}$ and $\widehat{q}_m^{(j)} := \frac{\widehat{\theta}_m^{(j)}}{\sum_{k\in T_m}\widehat{\theta}_m^{(j)}}$ denote the true and estimated relative within gene transcript expression levels and also $T_m := \{k$: transcript k in same gene as transcript m, $k = 1,\dots,M\}$, $m = 1,\dots,M$ denotes the set of transcripts belonging to the parent gene of each transcript.

WGE-Inter: $\sum_{i<j}\frac{2}{J(J-1)}\sum_{m=1}^{M}\frac{1}{M}\sum_{m=1}^{M}|\widehat{q}_m^{(i)} - \widehat{q}_m^{(j)}|$.

In addition we provide in Figure 10 the ranking of methods when the within gene estimates are computed according to the TPM measure, instead of θ. Figure 11 displays a comparison of ranking of methods when using only a subset of highly expressed transcripts.

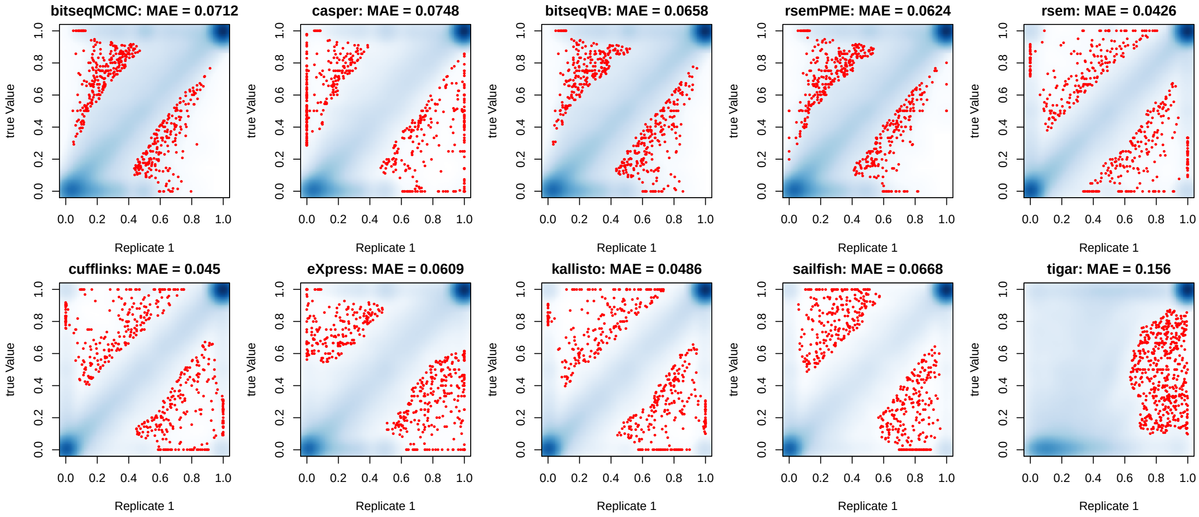

Fig. 7. Scenario 4: Scatterplots of within gene estimates for one replicate of the simulated data versus the true values. The blue color corresponds to a smoothed color density representation of the scatterplot and the red color emphasizes points from the lowest regional densities.

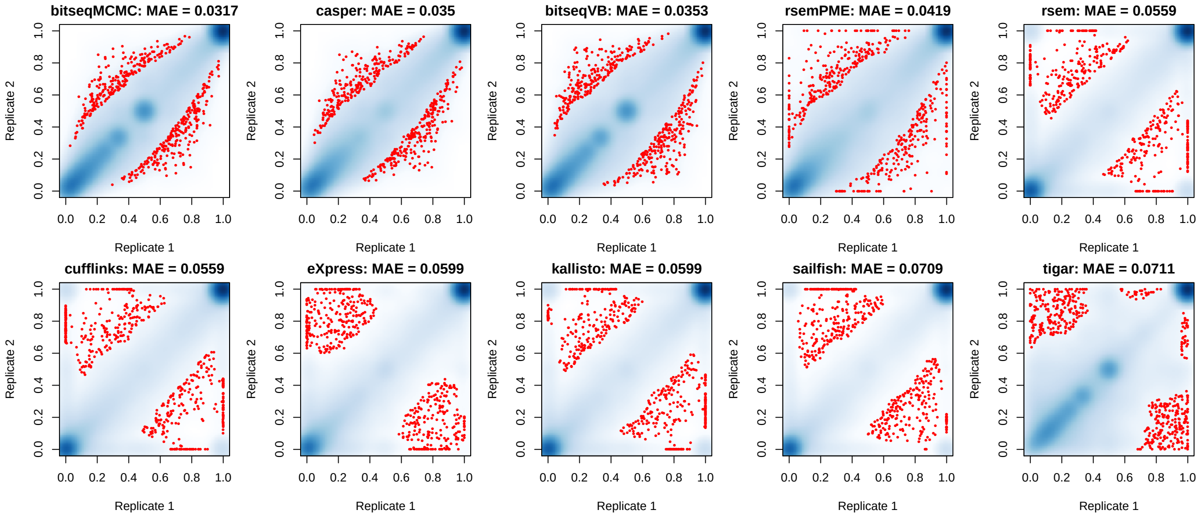

Fig. 8. Scenario 4: Scatterplots of within gene estimates for one pair of replicates from the simulated RNA-seq reads. The blue color corresponds to a smoothed color density representation of the scatterplot and the red color emphasizes points from the lowest regional densities.

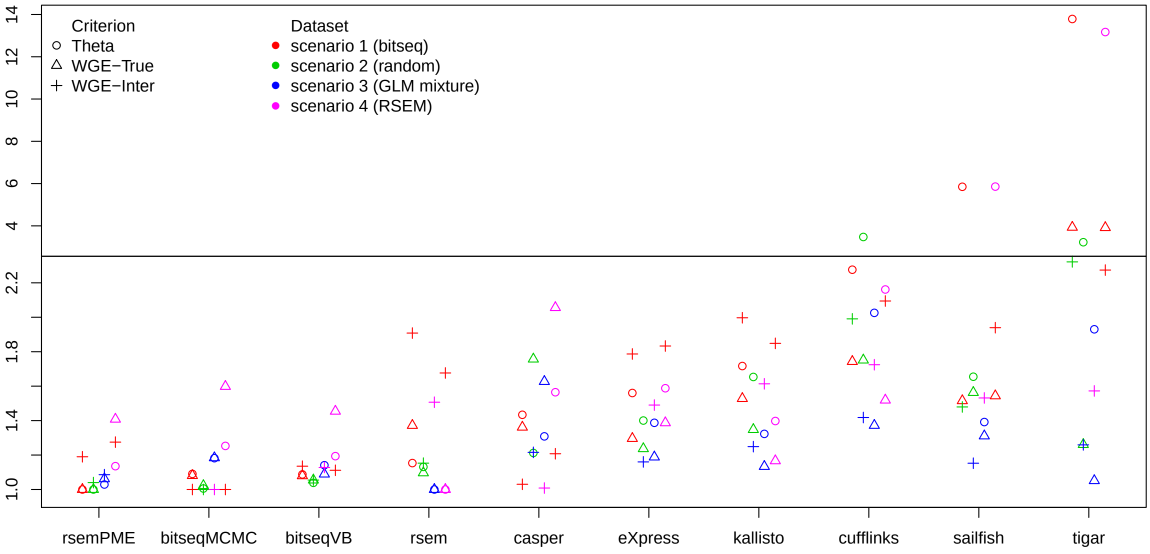

Fig. 9. Ranking of methods for five replicates of simulated RNA-seq reads. WGE-Inter: inter-replicate consistency of within gene estimates, WGE-True: within gene estimates compared to the true values and Theta: estimated relative transcript expression compared to the true values. Scores have been normalised by dividing by the min score per criterion.

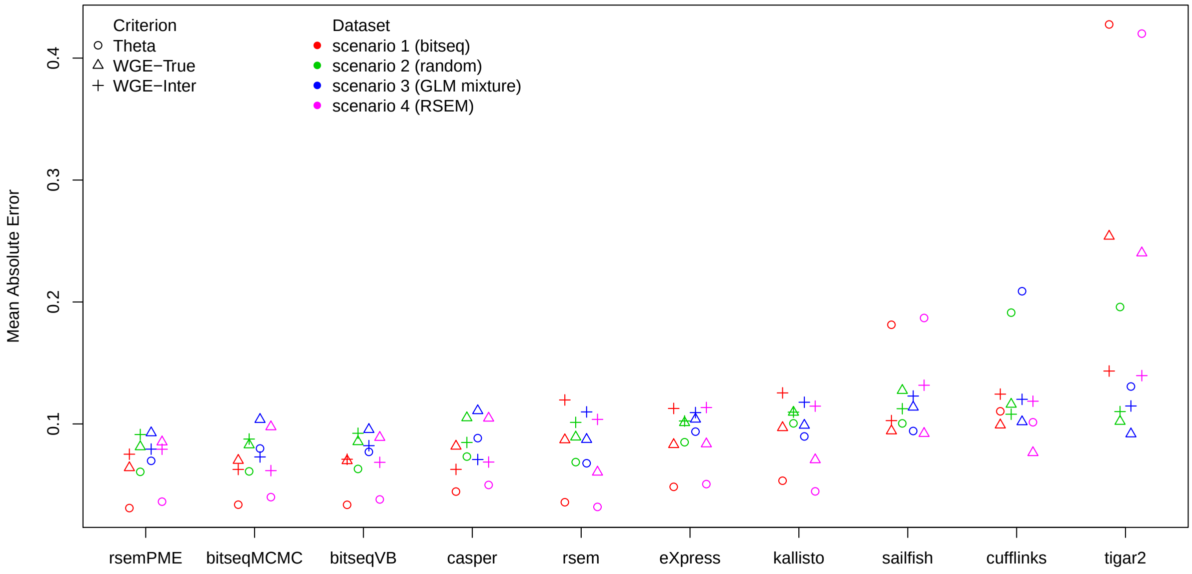

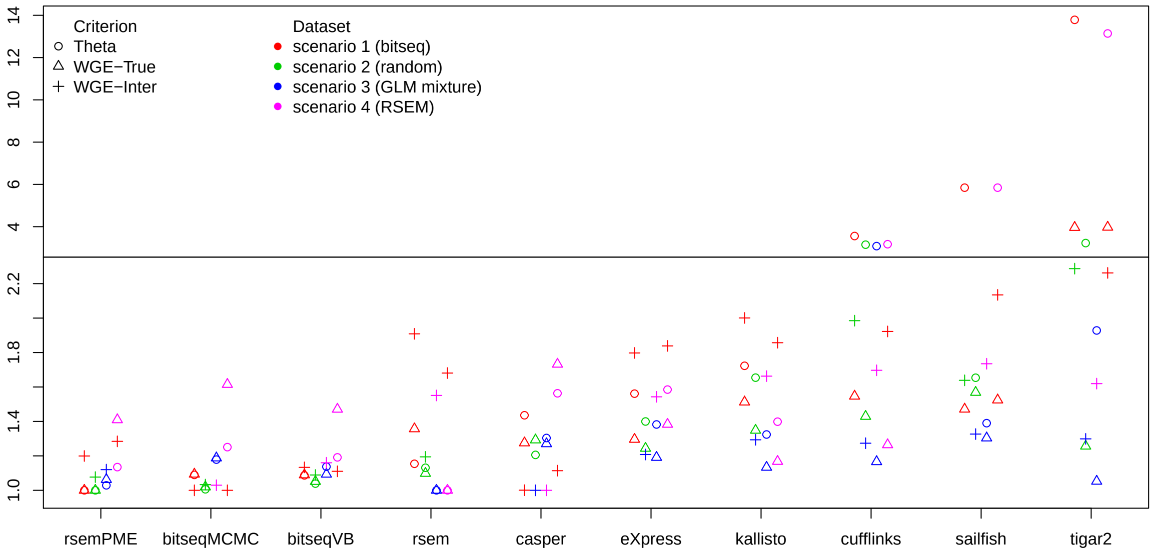

Fig. 10. Ranking of methods when using the TPM instead of theta for the within gene estimates (up: normalization according to unity sum, down: normalization by dividing by the min).

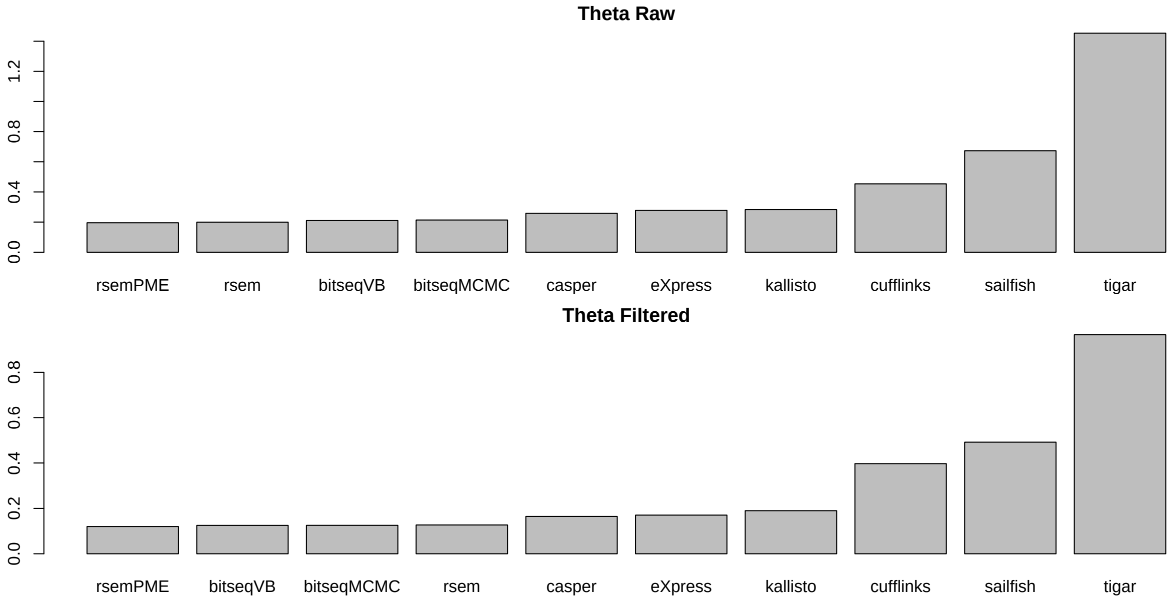

Fig. 11. Ranking of methods according to Theta when using all expressed transcripts (up) and only the ones with at least 50 reads per replicate (down).

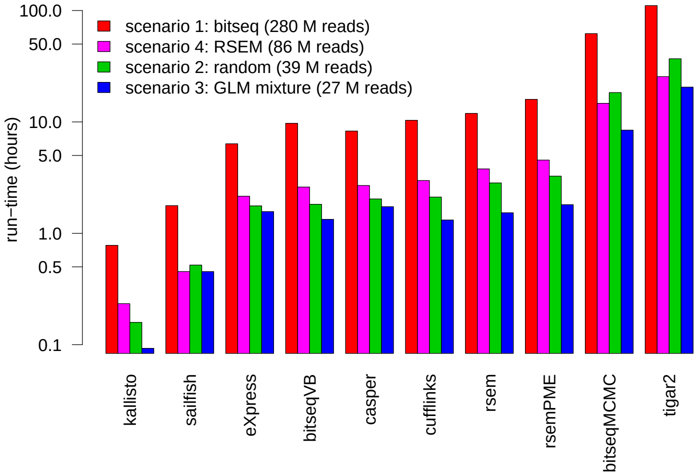

Fig. 12. Runtime per method including the time needed for the alignment procedure.
